# Immunotherapy for early triple negative breast cancer: research agenda for the next decade

**DOI:** 10.1038/s41523-022-00386-1

**Published:** 2022-02-18

**Authors:** Paolo Tarantino, Chiara Corti, Peter Schmid, Javier Cortes, Elizabeth A. Mittendorf, Hope Rugo, Sara M. Tolaney, Giampaolo Bianchini, Fabrice Andrè, Giuseppe Curigliano

**Affiliations:** 1 Division of New Drugs and Early Drug Development, European Institute of Oncology IRCCS, Milan, Italy; 2 Department of Oncology and Hemato-Oncology, University of Milan, Milan, Italy; 3 Barts Cancer Institute, Queen Mary University of London, London, UK; 4 Oncology Department, International Breast Cancer Center (IBCC), Quiron Group, Barcelona, Spain; 5 Medica Scientia Innovation Research (MedSIR), Barcelona, Spain; 6 Breast Cancer Research Program, Vall d´Hebron Institute of Oncology (VHIO), Barcelona, Spain; 7 Universidad Europea de Madrid, Faculty of Biomedical and Health Sciences, Department of Medicine, Madrid, Spain; 8 Division of Breast Surgery, Department of Surgery, Brigham and Women’s Hospital, Boston, MA USA; 9 Breast Oncology Program, Dana-Farber Cancer Institute, Boston, MA USA; 10 University of California San Francisco, UCSF Helen Diller Family Comprehensive Cancer Center Precision Medicine Cancer Building, San Francisco, CA USA; 11 Medical Oncology, Dana-Farber Cancer Institute, Boston, MA USA; 12 Harvard Medical School, Boston, MA USA; 13 Department of Medical Oncology, IRCCS Ospedale San Raffaele, Milan, Italy; 14 Department of Medical Oncology, Gustave Roussy, Villejuif, France

**Keywords:** Drug development, Breast cancer, Immunoediting

## Abstract

For decades, the systemic treatment of localized triple negative breast cancer (TNBC) has exclusively relied on chemotherapy. Recent advancements, however, are rapidly reshaping the treatment algorithms for this disease. The addition of pembrolizumab to neoadjuvant chemotherapy has indeed shown to significantly improve event-free survival for stage II–III TNBC, leading to its establishment as new standard of care in this setting. This landmark advancement has however raised several important scientific questions. Indeed, we desperately need strategies to identify upfront patients deriving benefit from the addition of immunotherapy. Moreover, the best integration of pembrolizumab with further recent advancements (capecitabine, olaparib) is yet to be defined. Lastly, extensive efforts are needed to minimize the impact on patients of immune-related adverse events and financial toxicity. The next decade of clinical research will be key to overcome these challenges, and ultimately learn how to optimally integrate immunotherapy in the treatment landscape of TNBC.

## Introduction

Triple negative breast cancer (TNBC) has long been a challenging disease to treat due to its aggressive behavior and the lack of actionable targets^1^. It is commonly diagnosed at a younger age compared with other breast cancer (BC) subtypes, and has a poor prognosis in case of metastatic relapse, with a median overall survival (OS) of less than two years^2^. Thus, intensive treatment strategies have been developed, to reduce the odds of recurrence after tumor removal. Poly-chemotherapy remains the standard treatment for early TNBC, most often administered preoperatively to assess tumor sensitivity and adapt post-operative systemic treatment accordingly^3^. Indeed, patients with residual disease after neoadjuvant chemotherapy are at the highest risk of recurrence^4^ and derive a significant benefit from the addition of adjuvant capecitabine^5^. Conversely, only follow up is recommended for patients achieving pathological complete response (pCR) at surgery, despite the fact that the risk of relapse remains clinically relevant. Multiple novel agents have been tested in the last decades to improve the prognosis of early TNBC, with none entering clinical practice, except for the recent approval of adjuvant olaparib for the subset of high-risk TNBC patients harboring germline BRCA1 or BRCA2 pathogenic variants^6^. However, the emergence of cancer immunotherapy is now revolutionizing the way we treat this disease.

## The rise of immunotherapy for TNBC

Despite lacking canonical targets for biologic treatment, TNBC is characterized by a relatively high tumor mutational burden (TMB) compared to other subtypes of BC, a feature which has been linked with increased responsiveness to immunotherapy with immune-checkpoint inhibitors (ICIs)^7^. Indeed, checkpoint inhibition with atezolizumab (now withdrawn in the U.S.) and with pembrolizumab has been approved for advanced-stage, PD-L1 positive TNBC based on the improvement in outcomes observed when combined with frontline chemotherapy^8,9^. Notably, evidence suggest a superior efficacy of ICIs in TNBC when administered early in the disease course, possibly due to the progression of immune escape mechanisms during the advancement of disease^10,11^. From this perspective, there was strong rationale for ICI administration to the earliest possible time in the disease course, namely before surgical resection. Results from several randomized trials designed with this purpose are now available, igniting a rapid change of practice in early TNBC.

Of five main randomized trials testing the addition of an anti-programmed cell death-1 (PD-1)/programmed cell death ligand-1 (PD-L1) agent to neoadjuvant chemotherapy^12–16^, three showed an improvement in pCR rate with immunotherapy^12–14,17^ (Table 1). Furthermore, long-term analyses have recently also demonstrated a survival benefit with this strategy. First, long-term results of the randomized GeparNuevo phase 2 trial were presented at 2021 ASCO Annual Meeting: although the trial has not met its primary endpoint of improving pCR^15^, the addition of durvalumab to neoadjuvant chemotherapy for high-risk TNBC patients ultimately improved 3-year invasive disease-free survival (iDFS) from 76.9% to 84.9% (HR = 0.54, *p* = 0.0559) and OS from 83.1% to 95.1% (HR = 0.26, *p* = 0.0076)^18^. These results, although suggestive of a benefit, required confirmation, since the trial was not powered to detect survival differences. More recently, the ESMO Virtual Plenary presentation of the mature event-free survival (EFS) results from the KEYNOTE-522 trial brought key new data in this setting, demonstrating that adding checkpoint inhibition in the early stage setting does in fact improve long-term outcomes^19^.

**Table 1:** Features and outcomes of the main randomized chemo-immunotherapy trials in early-stage triple negative breast cancer.

Trial name	Phase	Primary endpoint	Population enrolled	Regimen	pCR outcome (95% CI), %	Survival outcomes
KEYNOTE-522	3	pCR and EFS in ITT	Untreated stage II–III TNBC patients (*n* = 1174)	Neoadjuvant TCb -> AC ± pembro, followed by pembro (or placebo) for 1 year after surgery	64.8% vs 51.2%; delta 13.6% (5.4–21.8), *p* < 0.001	3-year EFS 84.5% vs 76.8% (HR 0.63, 95% CI 0.48–0.82, *p* = 0.0003) 3-year DDFS 87% vs 80.7% (HR 0.61, 95% CI 0.46-0.82) 3-year OS 89.7% vs 86.9% (HR 0.72, 95% CI 0.51–1.02, *p* = 0.032)
Impassion031	3	pCR in ITT and in PD-L1 + patients	Untreated stage II–III TNBC patients (*n* = 333)	Neoadjuvant nabT -> AC ± atezo, followed by atezo (or placebo) for 1 year after surgery (capecitabine also allowed)	58% vs 41%; delta 17% (6–27), *p* = 0.0044	EFS HR 0.76 (95% CI 0.40–1.40) DFS HR 0.74 (95% CI 0.32–1.70) OS HR 0.69 (95% CI 0.25–1.87)
NeoTRIPaPDL1	3	EFS	Untreated stage II–III TNBC patients (*n* = 280)	Neoadjuvant nabTCb ± atezo followed by adjuvant anthracyclines after surgery	43.5% vs 40.8%; OR, 1.11 (0.69–1.79), *p* = 0.66	Pending
GeparNuevo	2	pCR in ITT	Untreated stage I–III TNBC patients (*n* = 174)	Neoadjuvant nabT -> AC ± durva followed by physician’s choice of adjuvant treatment after surgery	53.4% vs 44.2%; OR, 1.45 (0.80–2.63), *p* = 0.287	3-year iDFS 84.9% vs 76.9% (HR 0.54, 95% CI 0.27–1.09, *p* = 0.0559) 3-year DDFS 91.4% vs 79.5% (HR 0.37, 95% CI 0.15–0.87, *p* = 0.0148) 3-year OS 95.1% vs 83.1% (HR 0.26, 95% CI 0.09–0.79, *p* = 0.0076)
I-SPY2	2	pCR in ITT	Untreated stage II–III TNBC and HR+/HER2- BC patients (*n* = 107)	Neoadjuvant T - > AC ± pembro followed by physician’s choice of adjuvant treatment after surgery	60% (44–75) vs 22% (13–20) (TNBC patients)	EFS HR 0.60 (TNBC patients)

*AC* anthracyclines plus cyclophosphamide, *atezo* atezolizumab, *BC* breast cancer, *Cb* carboplatin, *DFS* disease-free survival, *DDFS* distant disease-free survival, *durva* durvalumab, *EFS* event-free survival, *HER2* human epidermal growth factor receptor 2, *HR+* hormone receptors positive, *iDFS* invasive disease-free survival, *nabT* nab-paclitaxel, *OR* odds ratio, *OS* overall survival, *pembro* pembrolizumab, *pCR* pathological complete response, *T* taxane (paclitaxel or docetaxel), *TNBC* triple negative breast cancer.

KEYNOTE-522 was a phase 3 trial in which 1174 stage II–III TNBC patients were randomized to neoadjuvant chemotherapy with paclitaxel-carboplatin followed by doxorubicin-cyclophosphamide, with or without the addition of pembrolizumab; after surgery, patients received adjuvant pembrolizumab (or placebo) for up to nine cycles. Primary endpoints were pCR rate and EFS in the intention-to-treat (ITT) population. pCR results were published in early 2020, showing that among the first 602 patients randomized in the study the addition of pembrolizumab significantly increased pCR rate in the ITT (64.8% vs 51.2%, delta 13.6%; 95%CI, 5.4 to 21.8; *p* < 0.001), and an initial trend toward an EFS improvement was also observed^12^. That trend became a clear, statistically significant difference at the last update of the study results, which included 1174 randomized patients: with 37 months of follow up, 15.7% of the patients in the pembrolizumab arm and 23.8% in the placebo arm have experienced an EFS event (HR = 0.63, *p* = 0.0003)^19^. Three-year EFS rate was 84.5% with pembrolizumab versus 76.8% with placebo, showing a striking similarity with iDFS results from GeparNuevo^18,19^. Most EFS events were distant recurrences, leading to a 3-year distant-progression or distant-recurrence free survival of 87% with pembrolizumab versus 80.7% with placebo (HR = 0.61) and a clinically relevant, although not statistically significant difference in 3-year OS (89.7% vs 86.9%, HR = 0.72, *p* = 0.032)^19^. Intriguingly, the analysis of pCR rates among all 1174 patients at the third interim analysis showed a smaller delta between arms compared to the first analysis (63% vs 55.6%, delta 7.5%, 95%CI, 1.6 to 13.4)^20^, highlighting that differences in pCR rates can effectively translate into meaningful EFS benefits with immunotherapy. The addition of pembrolizumab led to an increase in immune-related adverse events (irAEs), with a rate of grade 3-5 irAEs of 14.9% (vs 2.1% in the control arm) and 10.9% of the events leading to any drug discontinuation (vs 2.6% in the control arm)^19^. Based on these compelling results, on July 26, 2021 the Food and Drugs Administration (FDA) approved pembrolizumab for high-risk, early-stage TNBC in combination with chemotherapy as neoadjuvant treatment, and then continued as a single agent as adjuvant treatment after surgery^21^.

## Identifying responders to immunotherapy: current status and future perspectives

KEYNOTE-522 results prompted a rapid change in clinical practice, leading to the FDA approval of the first immunotherapy agent for early-stage TNBC. This landmark achievement, however, has raised a multitude of scientific questions, requiring a new set of prospective clinical trials.

Indeed, every effort should be dedicated to identifying responders to pembrolizumab upfront, in order to tailor immunotherapy addition upon risk of relapse and immunological background. In this framework, subgroup analyses in KEYNOTE-522 did not highlight any biomarker solidly predicting the benefit of pembrolizumab^19^. In particular, despite PD-L1 expression being an established predictive biomarker in the advanced setting, it did not differentiate responders from non-responders in the early setting, with both PD-L1-negative and PD-L1-positive patients deriving a benefit from pembrolizumab addition^19^. It is however important to mention that the PD-L1 threshold adopted in the subgroup analyses of KEYNOTE-522 (CPS ≥ 1) may not be the optimal one, since a threshold of CPS ≥ 10 is currently used for patients selection in the metastatic setting, warranting this additional analysis in the future. A consistent benefit with pembrolizumab was also observed regardless of tumor size, carboplatin schedule, age and performance status^19^. Lastly, despite node positive patients appeared to derive greater benefit in terms of pCR from pembolizumab addition^12^, no difference in terms of EFS benefit were observed depending on nodal status at the survival analysis^19^.

Although standard parameters did not help in selecting patients for immunotherapy, novel promising biomarkers have recently emerged in this field. For instance, the detection of circulating tumor DNA (ctDNA) is emerging as a relevant prognostic factor across oncological diseases, including BC^22,23^. In the I-SPY2 trial, early BC patients receiving neoadjuvant treatment achieved outstanding disease outcomes with chemotherapy if no ctDNA could be detected at baseline, raising the question if any treatment escalation is required in this population^24^. Moreover, the presence in the tumor bed of tumor infiltrating lymphocytes (TILs) was shown to harbor a strong prognostic value for early TNBC, with tumors enriched in TILs showing excellent long-term prognosis with (neo)adjuvant chemotherapy^25^ and even in the absence of treatments^26^. Of note, when assessed in GeparNuevo trial, the presence of TILs appeared to predict benefit both in the durvalumab-containing arm and in the placebo arm, questioning the use of this biomarker alone to select patients for immunotherapy. Gains in CD274 gene (which encodes for PD-L1) were recently found to be common in TNBC, and associated with benefit to maintenance durvalumab in the advanced setting, warranting the study of this biomarker in the early setting for its promising predictive value^27^. The expression of Major Histocompatibility (MHC)-II complex on tumor cells was also retrospectively found to identify TNBC patients deriving benefit from the addition of immunotherapy to neoadjuvant chemotherapy in the I-SPY2 trial^28^. Lastly, evidence regarding the role of MHC I loss in immune-evasion is emerging for multiple tumor types^29^, warranting additional study in the field of breast cancer.

Besides baseline biomarkers, one established dynamic biomarker, namely the achievement of pCR after neoadjuvant treatment, showed a critical value in KEYNOTE-522. Indeed, a major absolute benefit in terms of EFS was observed among patients not achieving pCR, with a 10% improvement in 3-year EFS (from 56.8% to 67.4%) for patients receiving pembrolizumab, whereas only a 2% difference was observed in those patients achieving pCR. This finding - together with the results of GeparNuevo showing survival outcomes similar to KEYNOTE-522 with immunotherapy administered only before surgery^18^—support the experimental testing of strategies to de-escalate adjuvant immunotherapy in patients achieving pCR with chemo-immunotherapy. Nonetheless, until prospective evidence is available, current standards of care should include the adjuvant administration of pembrolizumab to all patients receiving it in the neoadjuvant setting without experiencing concerning irAEs. Moreover, when comparing EFS curves from patients achieving pCR in the two arms, it is important to stress the fact that the addition of pembrolizumab led to more patients achieving pCR, ultimately enriching the population of patients achieving a favorable EFS.

Noteworthy, extensive efforts will also be required to expand access to pembrolizumab to populations which were not included in KEYNOTE-522 but which are likely to derive benefit. Such is the case of ER-low patients (ER 1–9%), a population that was excluded from KEYNOTE-522 since not formally meeting the definition of TNBC, but which shares biology and dismal prognosis with canonical TNBC^30,31^, and could theoretically share the same benefit from the addition of immunotherapy.

## Integrating immunotherapy into an expanding arsenal of treatment options

One additional major challenge emerging from KEYNOTE-522 data is the optimal integration of pembrolizumab with other practice changes happening in the last few years. Indeed, since the design and initiation of the trial, two drugs have shown to significantly improve outcomes for TNBC patients at the highest risk of relapse, namely those not achieving pCR after neoadjuvant treatment^4^. First, the addition of 6-8 cycles of capecitabine in this high-risk cohort of patients showed to relevantly improve DFS and OS in the CREATE-X trial, rapidly reshaping treatment guidelines for TNBC^5^. More recently, the addition of one year of olaparib for BRCA1- or BRCA2-mutated TNBC patients not achieving pCR after neoadjuvant treatment showed a benefit in DFS and an initial trend in OS improvement in the OlympiA trial^6^. Neither of these two drugs were allowed in the KEYNOTE-522 trial, where all patients in the study arm received pembrolizumab alone as adjuvant treatment, regardless of residual disease at surgery^12^. Nonetheless, pragmatism is warranted in clinical practice, in order to derive the maximum benefit from the currently available therapies. In this framework, adjuvant treatment for patients with residual disease may be tailored according to residual cancer burden, overall recurrence risk and germline BRCA status. Therefore, for those patients appearing at the highest risk of recurrence, the addition of adjuvant capecitabine to pembrolizumab is reasonable; adjuvant olaparib ± pembrolizumab could be instead considered for high-risk BRCA-mutant patients. For both regimens we have indeed available data suggesting the safety of combining pembrolizumab with either capecitabine^32^ or olaparib^33^. Conversely, for patients with low residual cancer burden and low overall risk of recurrence, continuing pembrolizumab alone may be a reasonable option, if no concerning immune-related toxicities were experienced during neoadjuvant treatment.

Important new data has also recently emerged on neoadjuvant chemotherapy for TNBC. The BrighTNess trial, assessing the addition of veliparib plus carboplatin or carboplatin alone to standard neoadjuvant chemotherapy for high-risk stage II–III TNBC, has previously shown that the addition of carboplatin (but not veliparib) to anthracyclines and taxanes significantly improve pCR rates^34^. Survival results from this trial were recently presented at ESMO Congress 2021: the addition of carboplatin significantly improved 4-year EFS (79.3% vs 68.5%, HR = 0.57, 95%CI 0.36‒0.91, *P* = 0.018), whereas no benefit was observed with the addition of veliparib^35^. These results appear to confirm a long-term benefit of adding carboplatin, although it’s still unclear whether the same benefit is retained when adding ICIs: indeed, a survival benefit was also observed in the GeparNuevo trial, which did not include carboplatin in the neoadjuvant regimen^18^. In this setting, the inclusion of carboplatin appears reasonable in fit, high-risk, stage II–III TNBC patients, but new research efforts to clarify the need for platinum in the presence of pembrolizumab are urgently required, to understand if more flexibility is acceptable regarding the backbone chemotherapy regimen. Similarly, efforts will be needed to clarify if there is any role for associating dose-dense chemotherapy regimens to immunotherapy, based on the benefits observed with this strategy in prior trials^36^.

## The flip side of the coin: immune-related toxicities and accessibility issues

The price of improving patients’ outcomes with the addition of immunotherapy is the risk of irAEs beyond the toxicities of traditional chemotherapy^37^. The most common irAEs observed in KEYNOTE-522 were infusion reactions (18%), thyroid impairment (15.1%, hypothyroidism; 5.2% hyperthyroidism), skin toxicities (5.7%), pneumonitis (2.2%), hypophysitis (1.9%), colitis (1.7%) and hepatitis (1.4%) in combined neoadjuvant and adjuvant phases. Importantly, some of these are expected to be irreversible^38^, permanently conditioning the quality of life of patients in this curable setting. Additionally, concern on the impact of immunotherapy on fertility exist, particularly since TNBC often occurs in pre-menopausal patients^39^. In this regards, appropriate training of clinicians in the early identification and management of irAEs will be key for the mitigation of immunotherapy side effects. Concomitantly, these risks should be discussed with patients upfront, to provide a clear overview of the risks/benefits balance of adding immunotherapy to chemotherapy for the treatment of their tumor.

Besides immune-related toxicities, another major issue is the economic challenge represented by implementing high-priced drugs in the treatment algorithm of TNBC. Differences in the care and outcomes of patients with cancer arise or worsen with the discovery of new and more effective approaches to cancer treatment. By leading to the FDA approval of immunotherapy in the curative setting for TNBC, KEYNOTE-522 established itself as the archetype of the rapidly expanding use of immunotherapy across the spectrum of disease stages and as the epitome of the emerging disparities in access to these highly effective, but expensive, treatments^40^ Furthermore, clinical guidelines for BC often fail to appropriately consider health-system context or to provide adaptable guidance and are often incoherent with national cancer policies^41^. As a result, the design and implementation of effective and integrated multilevel interventions will be required to reduce barriers to equal access to immunotherapy and to equitably provide patients with the opportunity for longer and better-quality survival^40^.

## Research agenda for the next decade

**Fig. 1:**
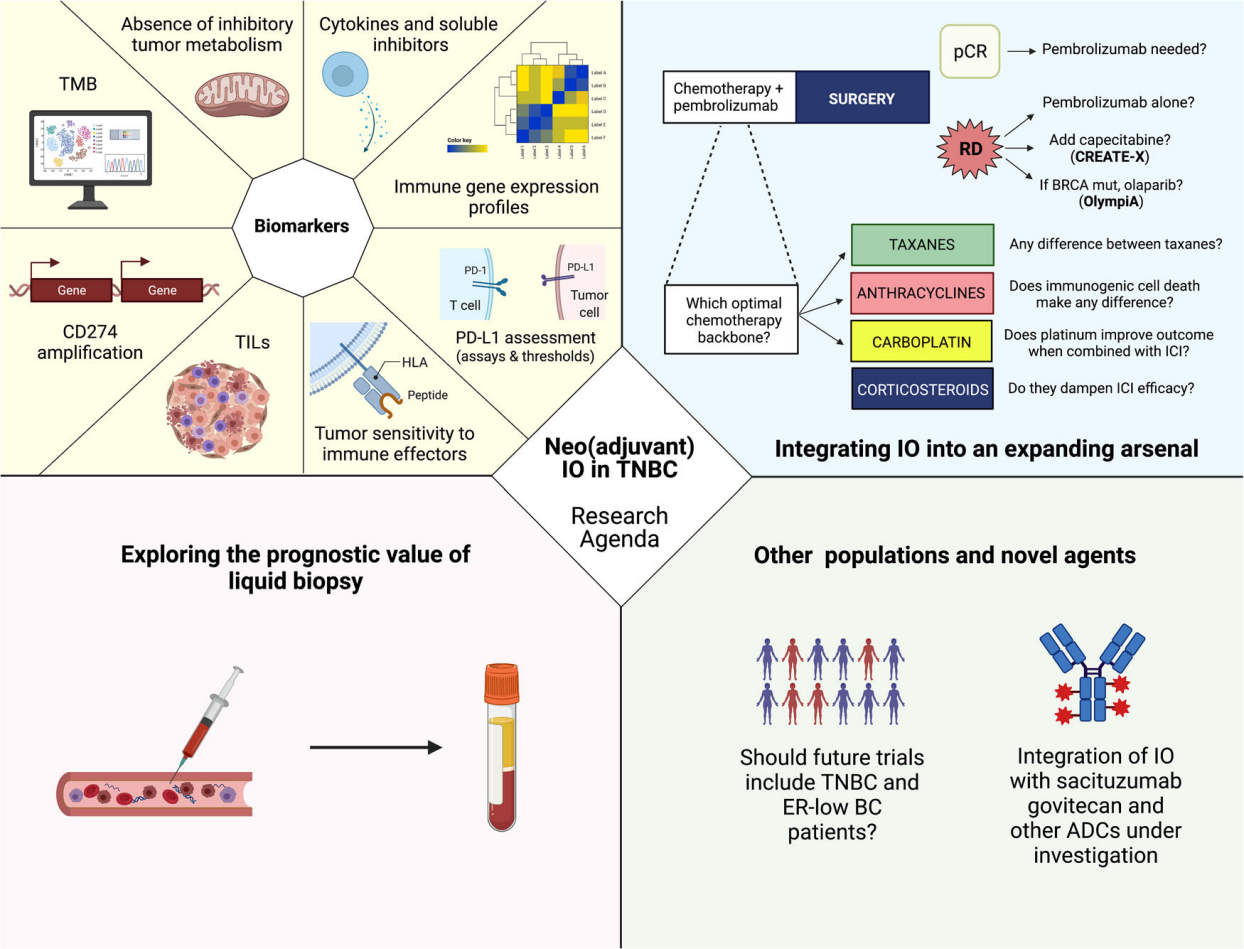
Next decade research agenda for neo(adjuvant) immunotherapy in TNBC. Abbreviations: IO, immunotherapy, TNBC, triple negative breast cancer; TMB, tumor mutational burden; ADC, antibody-drug conjugate; ER, estrogen receptor; CD, cluster of differentiation; TILs, tumor infiltrating lymphocytes; PD-L1, Programmed death-ligand 1; HLA, human leukocyte antigen; PD-1, Programmed cell death protein 1; A, adenosine; T, thymine; C, cytosine; G, guanine; BRCA, BReast CAncer gene; EFS, event-freee survival; RD, residual disease; me1, mono-methylated form; BC, breast cancer. Created with biorender.com.

The introduction of immunotherapy marks a revolution in the treatment of early-stage TNBC. KEYNOTE-522 has shown that, by unleashing anti-cancer immune responses through ICIs, long-term benefits can be obtained for the treatment of this aggressive BC subtype. However, it represents a starting point rather than a finish line, and additional efforts will be required precisely implement immunotherapy for the treatment of TNBC (Fig. 1).

First, biomarkers are desperately needed to optimally identify patients requiring the addition of ICIs to chemotherapy. In this regard, although PD-L1 expression determined with the 22C3 assay did not appear to differentiate responders in KEYNOTE-522, further immune-based biomarkers should be deeply investigated, including different assays and thresholds of PD-L1 expression, the presence of TILs, TMB, the value of CD274 amplifications, MHC-II expression, and immune gene expression profiles. Of note, an integration of these features into a comprehensive immunogram could potentially overcome the limitations of single biomarkers^42,43^.

Second, strategies should be investigated to dynamically adapt treatment according to the achievement of pCR. This is indeed among the strongest prognostic factors available in TNBC^4^, and it is reasonable to test differentiated treatment strategies for patients with and without residual disease after neoadjuvant treatment^44^. Trials should test the actual need for adjuvant immunotherapy for patients achieving pCR after chemotherapy plus pembrolizumab, as well as the optimal integration of immunotherapy with post-neoadjuvant capecitabine and olaparib for patients with residual disease. Moreover, trials testing the addition of ICIs for patients not achieving pCR to chemotherapy alone (e.g. A-BRAVE trial - NCT02926196; SWOG S1418/BR006 trial - NCT02954874) are ongoing, and may allow to understand if even an adjuvant-only administration of immunotherapy could exert clinical benefits in this challenging population. Besides the achievement of pCR, an emerging tool in this setting which deserves deeper investigation is ctDNA detection, which showed solid prognostic value in BC^45^ and other cancer histologies^22^. The cTRACK-TN trial (NCT03145961) is currently investigating the benefit of a tailored escalation of treatment with pembrolizumab for early TNBC patients with detectable ctDNA, and will provide precious data in this field of research.

Third, efforts should be invested in the expansion of neoadjuvant ICIs to other populations of patients potentially deriving benefit from this strategy. As mentioned above, ER-low (1–9%) patients appear biologically very similar to TNBC, with nearly 90% of these tumors harboring a basal-like intrinsic subtype^30^. Prognosis of these patients is also analogous to that of TNBC, highlighting the need for better treatments for this subgroup^30,31^. Future immunotherapy trials in TNBC should include this population of patients, to clarify if they derive the same benefit from the addition of ICIs to chemotherapy.

Fourth, novel active agents are emerging for the treatment of TNBC and could provide an opportunity for a de-escalation of traditional chemotherapy. In particular, the anti-TROP2 antibody-drug conjugate sacituzumab govitecan has recently shown to improve survival of TNBC patients in the advanced setting^46^, and it is currently being investigated in the early setting, including in combination with immunotherapy in the ASPRIA trial, where adjuvant sacituzumab govitecan in combination with atezolizumab is given to TNBC patients with residual disease after neoadjuvant treatment and detectable ctDNA. Results from this and additional studies will tell us if better outcomes can be achieved with the introduction of a targeted delivery of chemotherapy in TNBC.

## Conclusion

The improvement in outcomes provided by the addition of pembrolizumab to chemotherapy represents a landmark point for the treatment of early-stage TNBC. As for every major scientific advancement, these results raise a multitude of important questions, and a new set of prospective clinical trials will be required in the next decade to optimally tailor the administration of immunotherapy. This should be accompanied by a strong commitment in biomarker discovery and extensive effort devoted to the mitigation of both immune-related and financial toxicities, in order to achieve the safest possible implementation of immunotherapy for patients with a diagnosis of TNBC.

### Reporting summary

Further information on research design is available in the Nature Research Reporting Summary linked to this article.

## Supplementary information

Reporting Summary

## Data Availability

Not applicable.
